# Strategies for Embedding Prediction Models in Clinical Decision‑Making Workflows

**DOI:** 10.7759/cureus.101185

**Published:** 2026-01-09

**Authors:** Tope Amusa, Deborah Okunola, Osayimwense Izinyon, Abdulazeez Alabi, Olajide Akinpeloye

**Affiliations:** 1 Mathematics and Statistics (Biostatistics), Georgia State University, Atlanta, USA; 2 Statistics, Western Michigan University Homer Stryker M.D. School of Medicine, Kalamazoo, USA; 3 Epidemiology and Medical Statistics, University of Ibadan, Ibadan, NGA

**Keywords:** ai and machine learning, clinical decision support, electronic health record (ehr), implementation science in public health, prediction models, workflow integration

## Abstract

Machine learning and statistical prediction tools proliferate across healthcare, yet the leap from development to sustained clinical impact remains elusive. This narrative review synthesizes empirical evidence on how prediction models have been embedded into routine decision‑making and what lessons can be drawn for implementation teams. Searches of PubMed, Embase, Web of Science, IEEE Xplore, and specialized informatics journals (2010-2025) identified studies describing the real-world deployment of multivariable prediction models and reporting implementation outcomes. Evidence centered on sepsis detection, deterioration, readmission, and emergency triage models. Embedding strategies ranged from interruptive pop‑ups and non‑interruptive dashboard displays to worklists and order‑set linkage. Successful deployments invested heavily in stakeholder co‑design, threshold selection, training, and performance monitoring. Comparative studies indicated that deployment of a deep‑learning sepsis model (COMPOSER (COnformal Multidimensional Prediction Of SEpsis Risk)) decreased in‑hospital mortality and improved guideline adherence relative to baseline. Major barriers included workflow misalignment, alert fatigue, lack of transparency, data quality issues, and insufficient governance structures. Few papers described ongoing monitoring. The evidence suggests that prediction models confer value only when embedded through carefully designed clinical decision support aligned with the “Five Rights” framework, supported by multidisciplinary governance and rigorous monitoring. Implementation teams should prioritize calibration and decision utility metrics over discrimination alone, establish model‑life‑cycle governance, and integrate clinician training to build trust.

## Introduction and background

Clinical prediction models translate patient data into risk estimates or diagnostic probabilities. These models may derive from logistic regression, gradient‑boosted trees, or deep neural networks and are distinct from simple rule‑based alerts. Embedding refers to the integration of model output into specific decision touchpoints: who sees the prediction, when it appears, through which interface, and with what action options [1]. Without meaningful embedding, a score remains inactive and fails to impact care. The concept of decision support predates machine learning and has long been guided by the “Five Rights” principles: decision aids should provide the right information to the right person, in the right format, through the right channel, and at the right time [1]. These principles remain relevant for modern prediction models but require adaptation for complex risk scores and continuously updating algorithms. For instance, while the 'right time' is straightforward for a static rule-based alert, a deep-learning model that updates predictions every five minutes and forecasts sepsis hours before clinical presentation demands new considerations: when should the alert fire, how should evolving risk be communicated, and how can clinicians act on probabilistic outputs rather than binary flags?

The proliferation of machine learning publications has not translated into widespread, demonstrable clinical benefits. Hundreds of sepsis models have been described, yet successful integrations into routine clinical care are exceedingly rare [2], and a systematic review found that only approximately 10% of clinical prediction models undergo external validation [3]. When deployment occurs, performance often falls short of expectations because the environment shifts, data elements differ, or the model fails to integrate with local workflows. For example, early versions of commercial sepsis alerts within widely used electronic health record (EHR) platforms exhibited poor positive predictive value and provoked mistrust among clinicians [4]. Systematic reviews of artificial intelligence (AI) adoption have highlighted several barriers, including workflow disruption, inadequate integration, resource limitations, and insufficient training [5]. These sociotechnical factors have received less attention than algorithmic accuracy, yet they largely determine whether a prediction model influences decisions.

The aim of this narrative review is to integrate empirical evidence on strategies for embedding prediction models into clinical decision‑making and to examine how these strategies affect implementation and clinical outcomes. We focus on multivariable models (statistical or machine learning) that have been operationally deployed to influence decisions in hospital or emergency settings. Key outcomes include process changes (e.g., antibiotic timing), clinician uptake, alert adherence, patient outcomes, workload, trust, and equity. We exclude purely retrospective validations and simulation studies. By synthesizing findings across sepsis detection, deterioration, readmission, and triage models, and by framing the discussion through implementation and sociotechnical lenses, the article provides practical guidance for informatics teams tasked with deploying predictive analytics.

## Review

Methods

This review employed a narrative approach because the included studies varied substantially in design (randomized trials, before-and-after evaluations, qualitative studies), measured outcomes (mortality, alert adherence, time to treatment), and clinical contexts, precluding meaningful pooled analysis. Searches were performed in PubMed/MEDLINE (Medical Literature Analysis and Retrieval System Online), Embase, Web of Science, IEEE Xplore, and the Journal of Medical Internet Research (JMIR) and Journal of the American Medical Informatics Association (JAMIA) databases for articles published between 2010 and May 2025. Keywords included “clinical prediction model”, “risk score”, “machine learning”, “clinical decision support”, “workflow”, “implementation”, “deployment”, and “EHR integration”. Reference lists of key papers were scanned for additional studies. Inclusion criteria comprised peer‑reviewed empirical reports describing the deployment of a multivariable prediction model into clinical workflows and reporting implementation processes or outcomes. Quality appraisal was performed narratively without a formal scoring tool, as none are validated for the heterogeneous study designs included. We assessed studies based on study design (prioritizing randomized and controlled designs), sample size, the completeness of implementation reporting, and their relevance to embedding strategies. This method emphasizes conceptual synthesis rather than exhaustive coverage, appropriate for a narrative review aimed at implementation guidance.

Results

Clinical Contexts and Model Types

The literature identified four dominant clinical domains for real-world prediction model deployment: sepsis/deterioration, hospital readmission, emergency department triage, and intensive care unit (ICU) deterioration. Sepsis detection accounted for the majority of empirical reports. Models used ranged from logistic regression and Cox proportional hazards to gradient‑boosted trees and deep neural networks. Deployment settings were predominantly large academic hospitals in high‑income countries; few reports came from community hospitals or low‑resource settings. Study designs included before-and-after evaluations, interrupted time‑series analyses, and pragmatic clinical trials.

One of the most extensively reported deployments is the Sepsis Watch program at Duke University, which analyzed 86 variables from 50,000 patient records containing 32 million data points [2]. The deep-learning model updates predictions every five minutes and can predict sepsis a median of five hours before clinical presentation. Implementation required substantial investment in infrastructure, stakeholder engagement, and training, and led to the first full-scale deep learning integration into routine care. Clinicians and data scientists co-designed the interface, which displays high‑risk patients on a mobile dashboard accessed by rapid response nurses. Following deployment in November 2018, Duke doubled its three-hour SEP-1 bundle (Severe Sepsis and Septic Shock Management Bundle) compliance [2]. This illustrates the substantial organizational effort required to operationalize advanced prediction models. Clinicians and data scientists co-designed the interface, which displays high‑risk patients on a mobile dashboard accessed by rapid response nurses. The process involved aligning stakeholders, defining roles, and establishing partnerships with research groups [2].

Another prominent example is the COMPOSER deep‑learning model for early sepsis prediction at the University of California, San Diego. Deployed as a nurse‑facing Best Practice Advisory (BPA) within the Epic EHR (Epic Systems Corporation, Madison, Wisconsin, United States), COMPOSER generated 1.65 alerts per nurse per month. Boussina et al. completed an analysis of 6,217 adult septic patients spanning 850 days (705 days pre‑intervention, 145 days post‑intervention from January 2021 through April 2023) and found that COMPOSER deployment was associated with a 1.9% absolute reduction in in‑hospital sepsis mortality (17% relative decrease), translating to 22 additional survivors during the five-month intervention period. Sepsis bundle compliance increased by 5 percentage points absolute (10% relative increase), and 72-hour Sequential Organ Failure Assessment (SOFA) scores improved by 4% [4]. The study highlights how a carefully designed, calibrated intervention can translate predictive power into measurable improvements in care processes and outcomes.

Studies of readmission and deterioration models often reported more modest or neutral impacts. Bedoya et al. studied National Early Warning Score (NEWS) implementation across 85,322 patients (42,402 pre-implementation, 42,920 post-implementation) at two Duke facilities over 12 months [6]. The system generated 175,357 best practice advisories, with some patients receiving over 100 alerts per 24 hours. Frontline nursing staff ignored 86% of alerts, likely reflecting low alert thresholds that triggered warnings for clinically stable patients and poor alignment with nursing workflow, and the intervention showed no impact on the primary outcome of ICU transfer or death (adjusted hazard ratios of 0.94 (95%CI 0.84-1.05) at the academic hospital and 0.90 (95% CI 0.77-1.05) at the community hospital). Evidence for triage models in emergency departments is emergent; preliminary reports describe the integration of machine‑learning triage predictions into triage nurse dashboards but lack robust outcome evaluation. Table 1 summarizes the designs, settings, comparators, and headline outcomes of the studies on deploying prediction models identified in this review.

**Table 1:** Characteristics of included prediction model deployment studies Note: Studies ordered chronologically by publication year. Headline estimates reflect primary implementation or clinical outcomes as reported. Studies with "—" in the Comparator column used pre-post or descriptive designs without explicit control groups. BPA: best practice advisory; CI: confidence interval; ED: emergency department; EHR: electronic health record; EWS: early warning system; ICU: intensive care unit; LOS: length of stay; PPV: positive predictive value; QI: quality improvement; RCT: randomized controlled trial; RR: rapid response

Study (author(s), year)	Design & number	Population/Setting	Comparator	Outcome & Metric	Headline Estimate	Follow-up	Notable Limits
Sendak et al., 2020 [2]	Implementation study; qualitative evaluation	ED patients; Duke University Hospital (academic)	Pre-deployment period	Clinician engagement; workflow integration	High uptake among RR nurses (qualitative)	Implementation phase	No quantitative uptake metrics; single-site
Boussina et al., 2024 [4]	Quasi-experimental; 705 days pre-intervention versus 145 days post-intervention	Hospitalized patients; UC San Diego (academic)	Pre-intervention baseline	In-hospital sepsis mortality; bundle compliance	1.9% absolute mortality reduction; 5% bundle compliance increase	850 days total	Before-after design; secular trends; 1.65 alerts/nurse/month
Bedoya et al., 2019 [6]	Before-and-after; 6 months	General ward patients, an academic hospital	Usual care	Alert override rate, ICU transfer, and mortality	175,000 alerts (86% ignored); no mortality effect	6 months	Massive alert burden; 100+ alerts/patient/day; eroded trust
Wong et al., 2021 [7]	External validation; retrospective	Hospitalized patients; multi-site (academic)	—	Positive predictive value; alert burden	18% alert rate; detected 7% of undertreated sepsis	Cross-sectional	Low PPV; high false-positive rate; subgroup analyses absent
Nguyen et al., 2022 [8]	Implementation report	Pharmacy-targeted interventions; hospital setting	—	Worklist utilization; intervention timing	Real-time ranking enabled timely pharmacy actions	Implementation phase	Limited outcome data; process focus
Liengsawangwong et al., 2016 [9]	Quality improvement	Sepsis patients; hospital setting	—	Order set linkage; sepsis bundle initiation	Direct link to the sepsis order set with a verification box	Not reported	Limited design details; conference report
Chanas et al., 2019 [10]	Before-after	Surgical ICU septic shock patients; single center	Historical controls (4.2h to antibiotics)	Time to antibiotic initiation	Reduced from 4.2h to 1.6h	Implementation period	Small sample; uncontrolled; team-based notification confounds
Sandhu et al., 2020 [11]	Qualitative study; semi-structured interviews	Clinicians using Sepsis Watch, Duke University	—	User experience: trust factors	High engagement; dashboard monitoring by RR nurses	Post-implementation	Qualitative only; selection bias in interviewees
Atkins et al., 2023 [12]	Systematic review; 18 studies	Sepsis patients with pharmacist involvement	Standard care	Time to antibiotics	Consistent reductions in time to antibiotics	Varies by study	Heterogeneous interventions; observational designs
Gallagher et al., 2020 [13]	Implementation study; continuous monitoring	Discharged patients; academic hospital (Epic EHR)	—	Readmission risk display; case manager utilization	25% of high-risk patients received interventions	Ongoing	Feasibility constraints; passive worklist display; no RCT
Rossetti et al., 2024 [14]	Cluster RCT (CONCERN trial); pragmatic	Ward patients; multi-site	Usual care	Mortality; length of stay	35.6% mortality reduction; 11.2% LOS reduction	Trial duration	Preprint; possible concurrent QI; robust design
Wong et al., 2024 [15]	Stepped-wedge trial	Hospital ward patients; multi-site	Pre-intervention	Protocol adherence; ICU admission; mortality	No change in ICU admission, LOS, or mortality	12 months	Null findings: digital EWS did not impact outcomes
Davis et al., [16]	Observational: performance monitoring	Sepsis alerts during the COVID-19 pandemic	Pre-pandemic period	Alert rate; calibration drift	43% alert increase; model temporarily deactivated	Pandemic period	Fairness drift; population shift; external validity loss

Embedding Patterns and Decision Touchpoints

**Figure 1:**
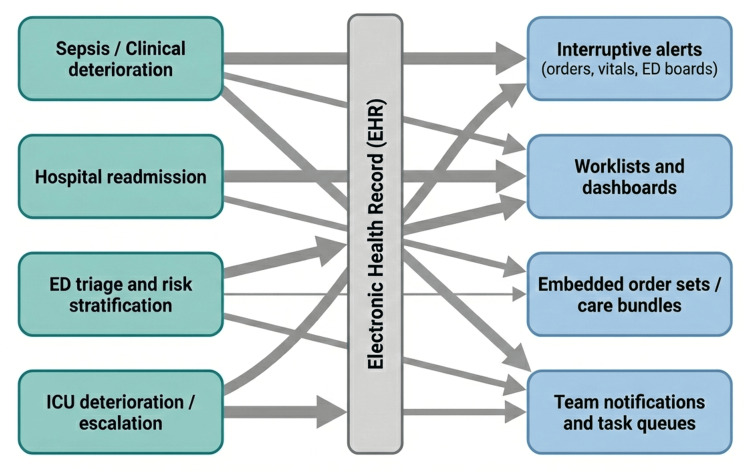
Deployment landscape for prediction models across clinical workflows Arrow thickness indicates which domain–workflow combinations appear most frequently in reported deployments. Image Credit: Tope Amusa (Author)

Five broad embedding patterns emerged from the review. Interruptive pop‑up alerts present the prediction as a modal window that requires acknowledgment before the user can continue, and they were the most common form of alert in real‑world deployments [17]. These alerts can prompt immediate action but risk causing alert fatigue and workflow interruption when thresholds are set too low. Non-interruptive displays show the score on flowsheets or dashboards, and clinicians can look at the information whenever they want. This feature cuts down on interruptions, but it can also make the information less visible and less useful [5]. Worklists and high‑risk lists group patients exceeding a risk threshold, enabling resource allocation and proactive rounding; for example, the PARADE (Patients at Risk for Adverse Drug Events) tool generated a real‑time, actionable worklist within the EHR for pharmacy teams and dynamically ranked patients for timely interventions [8]. Order‑set linkage triggers recommended orders or pathways when risk exceeds a threshold, such as sepsis best‑practice advisories that include a direct link to a sepsis order set requiring clinicians to verify a box to launch the order set [9]. Team‑based notifications send messages or tasks to specific clinicians (e.g., rapid response teams) via paging or in‑basket systems; some best‑practice advisories allow nurses to page residents, pharmacists, and charge nurses to convene at the bedside for sepsis management [10]. Figure 1 summarizes these deployments by mapping the dominant clinical domains to the workflow delivery patterns through which prediction models surface in the EHR.

Design choices within these patterns matter. Determining who receives the alert, nurses, physicians, pharmacists, or multidisciplinary teams, affects adoption. The timing of alerts, such as whether they occur at triage or after laboratory results, along with the frequency of updates, influences their relevance. User interface elements such as color coding, explanatory text, and the ability to snooze or override shape trust and workload. Studies rarely provide sufficient details for replication; descriptions of thresholds, update intervals, and user interfaces are often absent, hindering generalization.

Implementation Outcomes and Effects on Care

Implementation outcomes reported include alert firing frequency, acceptance or override rates, adherence to recommended actions, and sustainability over time. In the COMPOSER trial, which delivered about 1.65 alerts per nurse per month, nearly all alerts were acknowledged promptly and usually accepted [4]. Sepsis Watch reports described high engagement among rapid response nurses, though quantitative metrics were not published [11]. In contrast, some deterioration and readmission models generated dozens of alerts per clinician per shift: one National Early Warning Score (NEWS)‑based best‑practice advisory fired over 175,000 alerts in 12 months (some patients received more than 100 alerts per day), 86% of which were ignored, and other studies reported 49-96% override rates [6]. Such high alert burdens led to mistrust and, in some cases, deactivation of the alert systems [7].

The process outcomes include the time taken to administer antibiotics, adherence to sepsis bundles, transfers to the ICU, imaging orders, and timing of discharge. COMPOSER deployment improved sepsis bundle compliance by five percentage points and was associated with a reduction in in‑hospital mortality [4]. Machine learning early warning scores integrated into sepsis order sets or team‑based notifications have been associated with shorter time to antibiotics in uncontrolled before‑and‑after studies; for example, a sepsis best‑practice advisory that paged residents, pharmacists, and charge nurses reduced time to antibiotics from 4.2 hours to 1.6 hours in a surgical ICU cohort [10], and pharmacist involvement in sepsis response teams consistently decreased time to antibiotic administration [12]. Conversely, readmission models seldom change discharge planning or follow-up referral rates. Epic's readmission model at Duke identified high-risk patients on worklists discussed with case managers, but interventions were feasible for only approximately 25% of flagged discharges due to resource constraints and a lack of actionable pathways [13]. External validation of a widely implemented proprietary sepsis model revealed that while the model flagged 18% of all hospitalized patients, it detected only 7% of cases with undertreated sepsis, demonstrating low positive predictive value and high false-positive burden [7].

Clinical outcomes were usually reported without adequate rigor. COMPOSER demonstrated a 1.9% absolute mortality reduction, corresponding to 22 lives saved over five months [3], while the NEWS implementation study at Duke, enrolling 85,322 patients, showed no change in mortality or ICU transfers despite generating 175,357 alerts [5]. Observational analysis of deep learning models for ICU deterioration suggested reduced mortality and length of stay, but confounding by secular trends and concurrent quality‑improvement initiatives cannot be excluded. Some deployments reported increased hospital length of stay due to conservative decision-making when risk scores were high, illustrating potential unintended consequences.

Equity analyses were scarce. Only a handful of studies examined subgroup performance based on race, sex, or socioeconomic status; those that did found calibration drift and disparate false-positive rates, raising concerns about exacerbating existing disparities. Stakeholders emphasized the need to stratify monitoring dashboards by demographic variables to detect bias.

Sociotechnical Barriers, Facilitators, and Workflow Consequences

Barriers to successful embedding clustered around misalignment with established clinical workflows. Recent mixed-method and narrative syntheses of AI implementation in healthcare described poor integration with existing documentation, order entry, and communication processes, alongside concerns about workload, as dominant obstacles, and recommended alignment of model triggers and outputs with local work practices while preserving clinician autonomy [18-20]. Variations in documentation templates and risk-assessment workflows across units were associated with inconsistent model performance and challenged standardization of decision thresholds [18,21]. High alert firing rates contributed to alert fatigue, with frequent overriding or disabling of notifications in routine use, echoing long-standing concerns from clinical decision support evaluation [19,22]. Limited transparency regarding model logic, feature selection, and threshold setting weakened trust, particularly when predictions appeared indeterminate or clinically implausible. Data-quality problems, such as missing, delayed, or inconsistent vital signs and laboratory values, reduced performance, and demanded extensive preprocessing pipelines and ongoing monitoring of input streams [18,20].

Facilitators often engaged frontline clinicians early and continuously in the design of models and interfaces, ensured their explicit participation in threshold and alert-routing decisions, facilitated iterative refinement based on user feedback, and implemented structured training programs that focused on workflow integration and escalation pathways [23]. Integration of the Sepsis Watch deep-learning sepsis alert at Duke University, for example, relied on multidisciplinary co-design, a dedicated monitoring and feedback infrastructure, and repeated training sessions, which strengthened trust and clarified roles for nurses, physicians, and operational staff [2]. Qualitative evaluations of AI deployment in emergency, ward, and primary-care settings linked sustained trust to visible improvements in care processes and structured opportunities for clinicians to voice concerns, rather than to explanatory text alone [2,19,20]. Formal governance structures that defined accountability for deployment decisions, performance monitoring, recalibration, and retirement of models appeared to facilitate adoption and reduce ad-hoc local modifications [18,23].

Workflow consequences differed by delivery mode. Interruptive alerts embedded in order entry or vital-sign flowsheets supported urgent actions but increased cognitive load and contributed to alert fatigue in high-volume environments [19,22]. Non-interruptive dashboards and worklists reduced intrusion but required proactive engagement and clear allocation of monitoring responsibility [2]. Embedding prediction models into order sets streamlined the ordering process and increased adherence to recommended investigations or treatments, although the automatic triggering of defaulted bundles occasionally led to overuse [22]. Team-based notifications and shared worklists shifted responsibilities from individual physicians to distributed teams and required explicit hand-off rules to avoid duplication or omissions. Sociotechnical and behavioral frameworks such as the Behavior Change Wheel and derived tools, alongside Consolidated Framework for Implementation Research (CFIR) constructs, offer structured approaches to mapping organizational readiness, communication, and training needs. However, explicit use of such frameworks in reports of AI workflow integration remained uncommon within the studies underlying this review [20,22,23].

Governance, Monitoring, and Safety Signals

Evidence on post‑deployment monitoring and governance was limited. The Sepsis Watch team built custom dashboards to track real‑time model outputs and performance, but published few details [2]. Mayo Clinic described a platform for monitoring a production‑level machine‑learning model but noted that sustained operation required dedicated staff and infrastructure [24]. A scoping review of AI performance monitoring found a scarcity of evidence and guidance for the practical implementation of monitoring, identifying the importance of continuous surveillance, timely recalibration, and decommissioning when performance declines [25]**. **The review emphasized that acceptable performance at validation does not guarantee sustained adequacy and that models may require retraining, recalibration, or retirement as environments change.

**Figure 2:**
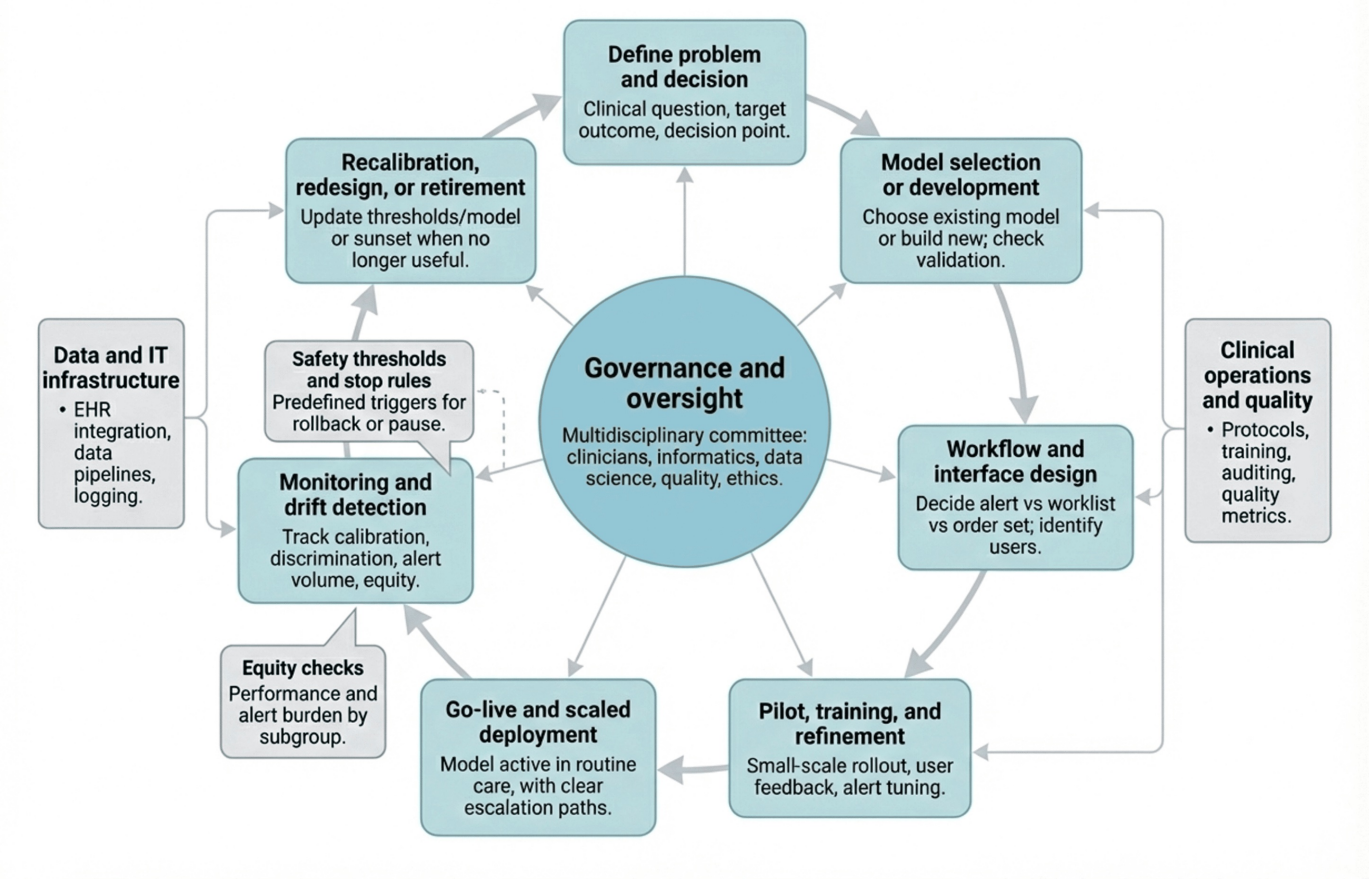
Lifecycle for embedding and governing clinical prediction models Solid arrows trace the clinical AI lifecycle from problem definition through deployment to recalibration, with governance at the center; side panels represent data/IT and clinical operations infrastructure, and dashed lines mark safety thresholds and equity checks around monitoring. Image Credit: Deborah Okunola (Author)

Regulatory bodies encourage monitoring but offer little specific guidance. The United States Food and Drug Administration and European authorities stress the importance of ongoing monitoring but do not specify methods for detecting performance drift [25]. Monitoring approaches include tracking input data distributions, model output distributions, feature importance, and downstream outcomes. Each method has limitations: indirect monitoring may miss clinically relevant drift, whereas direct outcome monitoring requires ground truth data that may not be readily available. Many health systems lack the infrastructure and expertise to implement these methods routinely. These deployment patterns, embedding strategies, and governance challenges reveal substantial variation in approaches and outcomes. Figure 2 depicts the lifecycle of embedding prediction models in care, linking problem definition, workflow design, piloting, scaled deployment, and ongoing monitoring to governance, safety thresholds, and equity checks.

Discussion

Real‑world deployments of prediction models reveal that only a minority of tools translate analytical accuracy into tangible improvements in care. This section interprets the evidence, compares successful and unsuccessful deployments, and considers factors that mediate impact.

Rethinking “Successful” Prediction Models: From Discrimination to Clinical Utility

Many studies report discrimination metrics, such as the area under the receiver-operating curve; however, these indices alone do not determine whether a model will improve care. In the results, deep‑learning sepsis tools such as COMPOSER and Sepsis Watch produced measurable benefits: COMPOSER, implemented as a nurse‑facing advisory in an academic hospital, reduced in‑hospital sepsis mortality by 1.9 percentage points and increased sepsis bundle compliance by 5 percentage points [4]. Sepsis Watch integrated a web dashboard monitored by trained rapid‑response nurses and led to high clinician uptake [11]. By contrast, logistic regression early warning scores and static deterioration alerts frequently generated tens of thousands of alerts, were ignored in more than 80% of cases and did not improve ICU transfer or mortality rates [6]. A digital early warning system trial reported no change in time for ICU admission, length of stay, or mortality [15]. These contrasts underscore that discrimination and calibration are necessary but not sufficient; decision value, alert burden, timeliness, and process integration must also be assessed. Net‑benefit analyses and decision curves can contextualize predictive performance by weighing true‑positive alerts against false positives and the associated workload.

Mechanisms of Success and Failure

Successful deployments share design features beyond algorithmic sophistication. COMPOSER and Sepsis Watch were built with multidisciplinary co‑design, aligning risk scores with specific clinical roles and workflows, providing clear action pathways, and maintaining moderate alert volumes (1.65 alerts per nurse per month for COMPOSER [4]; Sepsis Watch trained a dedicated team of rapid response nurses to monitor a dashboard updated every five minutes across 86 variables from 50,000 training records [2]). Rapid‑response nurses monitored dashboards and communicated with physicians, ensuring that predictions translated into timely clinical evaluations [11]. In sepsis best‑practice advisories, team‑based notifications and pharmacist involvement shortened time to antibiotics, illustrating how embedding models within collaborative workflows improves process metrics [10,12]. By contrast, deterioration alerts such as the NEWS were simply overlaid on general ward workflows; the Duke NEWS implementation fired 175,357 alerts across 85,322 patients over 12 months, with some individuals receiving over 100 alerts per day, saturating clinicians and eroding trust [13]. With an 86% override rate and no measurable impact on ICU transfers or mortality (adjusted HR 0.94, 95%CI 0.84-1.05), the system exemplified how high alert burden without workflow integration undermines effectiveness [6]. Readmission scores built from discharge data were presented passively on worklists; they identified a high-risk quartile but failed to alter discharge planning or follow-up because interventions were not actionable or resourced. Emergency department triage models remain predominantly developmental; real‑world implementation studies point out that they require cross‑departmental collaboration and rigorous integration planning before deployment [26].

These patterns suggest that timing, workflow fit, alert volume, and user engagement determine impact. Models that predict events earlier than existing practices and are tied to specific actions (e.g., ordering antibiotics) are more likely to improve outcomes than models that produce risk scores without clear intervention pathways. Conversely, overfrequent, non-specific alerts drive alert fatigue, resulting in overriding or disabling decision support [6]. Furthermore, algorithm predictions may be confounded by clinician actions: including interventions such as vital‑sign checks in the model may create feedback loops, and before‑and‑after evaluations cannot disentangle effects of underlying secular trends from model impact [27]. Randomized evaluations, such as the CONCERN (Communicating Narrative Concerns Entered by Registered Nurses) cluster trial, offer stronger evidence by mitigating confounding; preliminary findings from this preprint suggest that an early warning system reduced mortality by 35.6% and length of stay by 11.2%, though these results await peer-reviewed confirmation [14]. Meta-analyses of AI-enabled early warning systems suggest that hospital length of stay may diminish, whereas ICU length of stay may increase, potentially due to the earlier detection of patient deterioration and subsequent admissions. These findings emphasize the importance of high‑quality study designs and careful interpretation of outcomes.

Implementation experiences are heterogeneous across clinical domains, settings, and model types. Sepsis and deterioration models dominate the literature, whereas few studies report deployment of readmission, emergency‑department triage, or ICU deterioration models. Over 80% of deployments occur in the United States, mainly in academic hospitals, with limited reporting from community- or resource-constrained settings [17]. Logistic‑regression approaches still outnumber machine‑learning or deep‑learning methods. This geographic concentration limits the generalizability of current implementation guidance: health systems in low- and middle-income countries often face different infrastructure constraints (intermittent connectivity, limited EHR functionality), workforce compositions (fewer specialist responders), and workflow contexts that may require fundamentally different embedding strategies. Models calibrated on high‑resource, academically staffed hospitals may not perform similarly, or may require substantial adaptation, in these settings. Some sepsis models saw performance deterioration and alert surges when transferred across institutions or during the COVID‑19 pandemic; one proprietary model experienced a 43% increase in alert rate during the pandemic, prompting temporary deactivation [16]. Reliability also depends on data quality and workflow characteristics; emergency‑department triage models require integration with triage protocols and recognition that population acuity and data availability differ across sites [26]. Future implementations should therefore prioritize local validation, recalibration, and context-specific adaptations to maintain safety and effectiveness.

Equity and Fairness

The evidence base offers little insight into how prediction models perform across demographic groups. A review of algorithmic bias in safety‑net health systems noted that fairness metrics vary by use case and that there is no single optimal definition; threshold adjustments can mitigate disparities but require careful evaluation [28]. Few deployment studies report subgroup analyses of calibration or alert burden; the widely deployed proprietary sepsis model produced 18% alerts across hospitalizations, yet only 7% of sepsis patients lacked timely antibiotics [7]. Moreover, shifts in patient mix during the COVID‑19 pandemic resulted in a 43% increase in sepsis alerts, prompting temporary deactivation of the model [16]. These examples highlight how performance and fairness can drift over time and across populations. Continuous equity monitoring, tracking calibration, alert burdens, and outcomes by race, age, gender, and comorbidity, and adjusting thresholds or recalibrating models when disparities emerge are essential to preventing unintended harm. At a minimum, future deployment studies ought to present model performance (discrimination and calibration) disaggregated by race, ethnicity, age, sex, and socioeconomic status; alert burden for each subgroup; and any differential effects on clinical outcomes. When sample sizes preclude stratified analysis, authors should explicitly acknowledge this limitation and describe plans for post-deployment equity surveillance.

Quality of Evidence and Methodological Limitations

Many deployment studies rely on before-and-after or interrupted time‑series designs, which are susceptible to confounding, secular trends, and regression to the mean. The early warning paradox underscores that including interventions in predictive models can introduce bias and that observational evaluations cannot fully attribute outcome changes to model use [27]. Only a handful of cluster randomized or stepped‑wedge trials, such as the CONCERN early‑warning system trial, provide rigorous evidence; even then, outcome improvements may reflect concurrent quality improvement efforts [14]. Meta-analyses and systematic reviews identify heterogeneity in effect estimates and show that many AI‑enabled early warning systems fail to impact mortality, while some may prolong ICU stays [29]. There are no clear reporting standards for deployment studies. Many articles leave out information about interface design, threshold selection, user training, and monitoring, which makes it difficult to replicate and generalize. Common methodological gaps include small sample sizes, inadequate adjustment for confounders, absence of contemporaneous control groups, and failure to account for clustering effects when alerts target multiple patients per clinician. These limitations caution against over‑generalizing positive findings and reinforce the need for standardized reporting and rigorous study designs. This review itself has limitations inherent to narrative methodology. Article selection was not guided by a predefined protocol, introducing potential selection bias. The absence of quantitative synthesis precludes precise effect estimates, and the heterogeneity of included studies limits direct comparisons across embedding strategies. Nonetheless, the narrative approach permitted integration of diverse study designs and implementation contexts that would be incompatible with formal meta-analysis.

Integrating Sociotechnical Factors

Beyond algorithms, adoption depends on sociotechnical dynamics such as perceived usefulness, ease of use, workload, and organizational culture. Clinicians in AI adoption studies prioritized integration with existing workflows and autonomy; lack of integration, excessive alerts, and opaque algorithms eroded acceptance. Awareness and training programs improved understanding and trust, especially when delivered through co‑design and informal communication networks. Resource constraints, limited informatics personnel, inadequate infrastructure, and competing priorities impeded implementation [5]. Transparent communication about model rationale, performance, and limitations, along with defined governance structures for monitoring and updating models, fosters accountability and mitigates the “responsibility vacuum” that can accompany automated recommendations. Sociotechnical frameworks and implementation theories offer tools to map these determinants and guide change, but they remain underused in deployment reports. Addressing these human and organizational factors is as crucial as optimizing algorithms when embedding prediction models into care pathways.

**Table 2:** Implications for implementation and informatics teams AUC: area under the curve; PPV: positive predictive value

Domain	Practical Implication	Example Actions	Possible Metrics
Problem selection	Focus on high-burden, high-actionability use cases	Prioritize sepsis/deterioration over low-impact scores	Baseline event rate, potential absolute risk reduction
Workflow design	Align alerts with real decision points	Route ED alerts to triage; ward alerts to charge nurse	Alert response time, % alerts acted on
Training & adoption	Treat deployment as change management	Structured onboarding, refreshers, and feedback loops	Training completion, user adoption rate
Monitoring & drift	Embed routine performance checks	Monthly audit of AUC, calibration, and alert volume	AUC, calibration slope, alerts per 100 patient-days
Equity & safety	Track subgroup performance and burden	Compare PPV/alert volume by age, sex, and race	Subgroup AUC/PPV, alerts per subgroup
Governance	Establish a standing oversight group	Multidisciplinary committee with the authority to pause	Number of reviews/year, decisions on recalibration/retirement

Future Recommendations

Research and practice should advance in several directions. First, it is necessary to conduct comparative studies that evaluate embedding patterns, including interruptive alerts, dashboards, and order-set linkage, using robust designs such as cluster randomized trials or stepped-wedge studies. Current evidence largely relies on before-and-after comparisons with potential confounding. Second, multi‑site pragmatic evaluations should assess generalizability across hospital types and health systems. External validation and transportability remain understudied; calibration may drift when models move across settings [25]. Third, reporting standards should expand to cover embedding details, thresholds, interface design, and monitoring practices. Extensions to TRIPOD‑AI (Transparent Reporting of a multivariable prediction model for Individual Prognosis Or Diagnosis, AI extension) and CONSORT‑AI (Consolidated Standards of Reporting Trials-AI extension) could include checklists for deployment studies. Fourth, equity‑focused research should measure performance across demographic groups and investigate interventions to mitigate bias. Finally, decommissioning and replacement of models deserve attention; criteria for retiring or switching models when performance declines or better options emerge should be defined.

## Conclusions

The potential of prediction models resides not in the algorithms per se, but in their integration into clinical workflows. Real‑world deployments show that models can improve outcomes when embedded through thoughtfully designed decision support, robust training, and continuous monitoring. Yet most deployments fall short due to workflow misfits, alert fatigue, a lack of transparency, and an absence of governance. Implementation teams should treat embedding design, monitoring, and governance as first‑class elements alongside model development. Key success factors identified in this review include multidisciplinary co-design with frontline clinicians, calibrated alert thresholds that balance sensitivity against alert fatigue, clear linkage to actionable interventions, and sustained governance structures for performance monitoring and recalibration. Only through disciplined integration can predictive analytics realize their potential to enhance patient care.
